# Structural and Dimensional Analysis by Computed Tomography of a Multi Geometric Template Manufactured by Fused Deposition Modeling

**DOI:** 10.3390/mi14101934

**Published:** 2023-10-15

**Authors:** Julian I. Aguilar-Duque, Sinue Ontiveros, Yolanda Baez-Lopez, Victor Manuel Juárez-Luna, Agustín Brau-Avila, Diego Tlapa

**Affiliations:** 1 Facultad de Ingeniería, Arquitectura y Diseño, Universidad Autónoma de Baja California, Carretera Transpeninsular Ensenada-Tijuana 3917, Ensenada 22860, Mexico; julian.aguilar@uabc.edu.mx (J.I.A.-D.); juarezv@uabc.edu.mx (V.M.J.-L.); diegotlapa@uabc.edu.mx (D.T.); 2 Facultad de Ciencias de la Ingeniería, Administración y Sociales, Universidad Autónoma de Baja California, Tecate 21460, Mexico; sinue.ontiveros@uabc.edu.mx; 3 Department of Industrial Engineering, University of Sonora, Blvd. Luis Encinas y Rosales S/N, Col. Centro, Hermosillo 83067, Mexico; agustin.brau@unison.mx

**Keywords:** fused filament fabrication, computed tomography, dimensional analysis, roughness analysis

## Abstract

As a consequence of the development of AM, strategies have been developed to optimize the printing process, which focuses on reducing manufacturing time, such as using genetic algorithms (GAs), among others. The effect caused by the modification of path patterns is an effect of interest in two aspects: dimensional assurance focused on the compliance of the dimensions of the components in comparison with the digital design of the components, and the structural composition and resistance that the printing process itself can generate. This paper aims to present the effect of optimizing the path of fused filament fabrication (FFF) equipment on the dimensional finish and structural quality of a multi-geometric component using computed tomography. For this purpose, a template composed of 23 geometric elements, printed using FFF technology and PLA as the base material, is used. The results show an 11% reduction in the total process time required to print the component. The effect on the dimensional precision of different geometric elements was identified. In addition, it was possible to ensure that the structural quality of the multi-geometric component was not affected by the modification of the path required by the printing process.

## 1. Introduction

### 1.1. Manufacturing

New production technologies, recent marketing strategies, and compliance with customer demands based on quality standards are the guidelines that govern the development of the manufacturing and service industries [1,2]. Faced with such business development guidelines and the concern of subsisting in a dynamic production system, the development of analyses that are focused on comparative indexes between market growth and the development of countries involved in manufacturing and service activities was triggered [3]. With the wide range of existing competitors, the pressure on the economy, and inflation, it is possible to forecast that the growth of the manufacturing and service industry will be 3.7% by 2025 [4,5,6].

Considering the growth forecast for the manufacturing and service industries, subtractive manufacturing (SM) and additive manufacturing (AM) have defined their market niches based on the production capacity of their technologies. On the one hand, MS focuses on mass production systems with smooth finishes on components, higher dimensional tolerance, and medium-to-large volume production runs [7]. On the other hand, MA focuses on small production runs, with the manufacture of complex designs, and mechanisms, or components that do not require very high mechanical requirements [8,9]. However, despite the clear market definition that MS and MA have determined, according to their capabilities, both compete to satisfy the most developed productive sectors shown in Table 1.

### 1.2. Additive Manufacturing

Defined as the process of layer-by-layer fabrication from a digital design [11], additive manufacturing (AM) has made inroads into the component manufacturing industry via design freedom, product customization, reduced tooling costs, and, in some cases, reduced costs associated with logistic activities [12,13,14].

Starting in 1980 with the filing of the first AM technology patent, AM evolved into 22 technologies by 2022, which are fused deposition modeling or fused filament fabrication (FDM or FFF); vat photopolymerization; stereo-lithography (SLA); digital light processing (DLP); continuous digital light processing (CDLP); programmable photopolymerization (P3); material jetting; PolyJet; nanoparticle jetting (NPJ); drop on demand (DOD); binder jetting, powder bed fusion; multi-jet fusion (MJF); selective absorption fusion (SAF); selective laser sintering (SLS); selective laser melting (SLM); electron beam melting (EBM); direct energy deposition; laser engineering bet shape (LENS); electron beam additive manufacturing (EBAM); and laminated object manufacturing (LOM) [15,16,17,18]. This rapid technological growth is evidence that AM is advancing, seeking to strengthen the range of production possibilities in the face of existing manufacturing systems.

According to Businesswire [10], Economics [13], and Nikitakos et al. [19], the significant development of AM technologies has changed the perspective regarding the future of manufacturing processes; one of these changes is generated by the feasibility of the development of complex design elements, which are manufactured in a single process compared to the set of processes and sub-processes that the same design would demand in an SM process. Another change is associated with the projected growth of 16% in the industrial and professional printer sector and 40% in desktop and personal computers. Finally, it is worth mentioning that the use of AM has reduced waste that is generally produced during SM processes.

The development generated by AM has had an impact on the aerospace [20,21,22], automotive industry [23,24,25], biomedical [26,27,28], and construction industries [29,30,31,32]. The capacity of AM technologies has also evolved in the direction of process analysis and correction during the printing process, as mentioned in Guo et al. [33], who used high-speed, high-energy in situ X-ray imaging to reveal dynamic changes in the melt pool and depression zone during laser scanning to improve the quality of the component, or in the result generated in real time by the research of Wang et al. [34], who declared using real-time spattering and plume behaviors instead of volumetric energy density. Moreover, this is observed in Sinclair et al. [35], who made use of in situ radiographic and ex situ tomographic methods to analyze pore interactions during multilayer builds. All these cases were used to determine the capacity of the process for generating dimensionally appropriate components.

Despite the above-mentioned advantages of AM versus SM, AM has been characterized as a low-volume production system that is unable to compete against SM, with quality defects that are associated with component repeatability and dimensional variations; moreover, depending on the process, AM lacks resistance to extreme operating conditions, such as temperature, compressive, tensile, torsional, and bending stresses [36,37,38].

### 1.3. Fused Filament Manufacturing

Fused deposition modeling (FDM) or fused filament fabrication (FFF) is one of the AM technologies that bases its process on the extrusion of thermoplastics. The production system starts with the component’s design, is coded by the printing equipment, and then proceeds to the printing process layer by layer [39].

According to Market Data Forecast [40], due to the ease of operating FFF equipment and the low cost it represents for users, FFF technology has become the AM technology with the most economic gains generated during the last decade, achieving a total revenue of USD 550 billion, and it is distributed in prototyping, production, proof of concept, market samples, art, education, and hobby applications.

In a competitive environment, it is a reality; this reality is evolving a series of factors that generate the introduction of new production methods, research efforts, new product development, and the discovery of new materials [41,42,43,44]. Despite the growth of FFF, the technology exhibits quality defects associated with structural deformation [45,46,47], dimensional deformation [48,49], low quality [50,51,52], and processing time [53,54], which represent an obstacle to the use of this technology in the fabrication of functional prototypes and the direct digital fabrication of objects intended for liquids and gases.

### 1.4. Optimization Algorithm

One of the strategies employed by the software developers of printing equipment focused on reducing dimensional defects and the surface finish generated by the FFF process, and the strategy consists of implementing and developing different programming algorithms. These algorithms are based on the findings obtained from their implementation in operating computer numerical control (CNC) equipment. Some practical algorithms for FFF are robot path optimization for machining [55], the genetic algorithm (GA) for the reduction in tool travel time without adding value to the component (tool air time) [56], and GA for the optimization of operating parameters and reduction in operating times [57,58]. Recently, Yodo and Dey [59] presented their proposal for multiobjective optimization based on evolutionary algorithms.

As mentioned, the use of GAs has evolved considerably in the optimization of CNC or extrusion tool paths. It is worth mentioning that hybrid methods focused on the reduction in operation times have also been used: for example, the combination of the neural network algorithm with the response surface algorithm focused on the optimization of parameters [60]; the combination of the hybrid particle swarm optimization with bacterial foraging optimization to optimize operating parameters [61]; artificial immune systems (AISs) and artificial neural networks (ANNs) [62]; GA with particle swarm optimization (PSO) [63]; or that of Ülker et al. [64], which report the combination of GA with AIS.

Specifically in FFF, GAs have been executed to improve the performance of this technology based on the operating parameters and the determination of their optimal operating values, as demonstrated by [65] with the parameter optimization model or [12,54].

There is no doubt that the FFF process represents an alternative for the development of 3D-printed components; however, the time necessary for the manufacture of components and the dimensional finishing of the printed features represents an opportunity for progress from several research aspects. Considering the principles of transport methods, it is possible to make use of routing models, in which the path of the tool (extruder) depends on two questions: The first one is directed to the curve to be traced, and the second one is directed to the direction in which the curve will be printed. The mathematical expression of this principle is shown as a function of two variables for decision-making:

$$X_{i}=\left\{\begin{array}{c} 1\;\mathrm{if}\;\mathrm{the}\;\mathrm{initial}\;\mathrm{vertex}\;\mathrm{of}\;\mathrm{arc}\;i\;\mathrm{is}\;v_{i1}\\ 0\;\mathrm{if}\;\mathrm{the}\;\mathrm{initial}\;\mathrm{vertex}\;\mathrm{of}\;\mathrm{arc}\;i\;\mathrm{is}\;v_{i2} \end{array}\right.$$

$$Y_{ij}=\left\{\begin{array}{c} 1\;\mathrm{if}\;\mathrm{the}\;i-\mathrm{th}\;\mathrm{traced}\;\mathrm{arc}\;{arc}_{j}\\ 0\;\mathrm{any}\;\mathrm{other}\;\mathrm{case} \end{array}\right.$$

With the variables described above, the model’s objective function focuses on minimizing the total time required to perform the run, precisely the time that does not add value to the component’s printing (airtime). The time required is proportional to the distance traveled between subsequent arcs, and this is described by the following function (Equation (1)):

(1) $$Min\;Z=\sum_{j=1}^{n}Y_{1j}\left[X_{j}a_{0j}+\left(1-X_{j}\right)a_{1j}\right]+\sum_{j=1}^{n}Y_{nj}\left[X_{j}a_{1j}+\left(1-X_{j}\right)a_{0j}\right]+\sum_{i=1}^{n-1}\sum_{j=1}^{n}Y_{ij}\left(\sum_{i=1}^{n}Y_{i+1,l}Z_{jl}\right)$$

where:

$$Z_{jl}=X_{j}X_{l}a_{jl}+X_{j}\left(1-X_{l}\right)b_{jl}+\left(1-X_{j}\right)X_{l}c_{jl}+\left(1-X_{j}\right)\left(1-X_{l}\right)d_{jl}$$

n is the total number of curves to be printed.

$$a_{ij}=distance\left(v_{i2},v_{j1}\right)$$

$$b_{ij}=distance\left(v_{i2},v_{j2}\right)$$

$$c_{ij}=distance\left(v_{i1},v_{j1}\right)$$

$$d_{ij}=distance\left(v_{i1},v_{j2}\right)$$

$$a_{0j}=distance\left(origin,v_{j1}\right)$$

$$a_{1j}=distance\left(origin,v_{j2}\right)$$

The use of constraints that avoid loops allows Tuker’s formulation to obtain a transport model with the following objective function (Equation (2)):

(2) $$Min\;Z=\sum_{i=1}^{n}\sum_{j=1}^{n}Y_{ij}D_{ij}$$

Subject to:

$$\sum_{j=1}^{n}Y_{ij}=1\;for\;i=1\;until\;n,$$

$$\sum_{i=1}^{n}Y_{ij}=1\;for\;j=1\;until\;n,$$

$$Y_{ij}=\left[0,1\right]$$

$$u_{i}-u_{j}+{py}_{ij}\leq p-1\;para\;i=1,\ldots n;\;j=1,\ldots ,n;\;i\neq j$$

With the objective function of the previous model, u_i_ denotes the variables, and p is the maximum number of nodes that the extruder must cover from its initial position until it finishes printing the layer. Subject to the principle of integer programming models, a sequence in which arcs are optimally visited is determined.

### 1.5. Computed Tomography

It is no secret that computed tomography (CT) has become an essential option for the analysis and development of manufacturing systems, the main reason being its ability to perform multiple analyses and the ability to observe inside the part in a relatively short time compared to traditional measurement techniques [66,67]. With a single scanner, it is possible to obtain a 3D model of the workpiece and perform analyses inside the part (even in areas that are not accessible to the eye): dimensional analyses and analyses of multi-material parts without the need to separate the workpiece to perform reverse engineering. The 3D model of the workpiece can also be used to perform simulations, all without the need to destroy or intervene with the part [68].

CT is increasingly used to analyze complex geometries and additive manufacturing processes. Part interior analyses and dimensional accuracy are these parts’ most critical quality control analyses. Khosravany and Reinicke [69] presented a summary of industrial and academic applications—and models created with different additive manufacturing processes or techniques with respect to different materials and shapes—in order to mainly analyze porosity and material density; these models included regular geometries or repetitive manufacturing patterns. On the other hand, Cho and Lee [70] presented the use of CT for the porosity and material density analyses of a dog-bone-shaped specimen printed using carbon-fiber-reinforced plastic. In [71], a 3DP cylindrical scaffold with pores of 1 mm fabricated via additive layer manufacturing was analyzed. In [72], the filtration performance of 3D-printed ceramic pellets is studied using CT in order to determine whether the forms and materials are in accordance with the original design.

In [73], a review of the use of X-ray computed tomography for additives are presented. In this paper, many different types of pieces are discussed, with most of them comprising simple geometries, and there are a few with a maximum of four different types of geometries.

In the report presented by Tkac [74], CT was used to analyze the porosity of a part based on the 3D-printed model of the lattice structure. This same part is used in [75] to perform mechanical structure analyses.

The common feature of the parts mentioned in previous paragraphs is that they are parts with basic geometries or repeated geometrical patterns. The objective of the present research study is to determine the dimensional finish and structural quality of a component composed of multigeometric elements.

## 2. Materials and Methods

**Figure 1:**
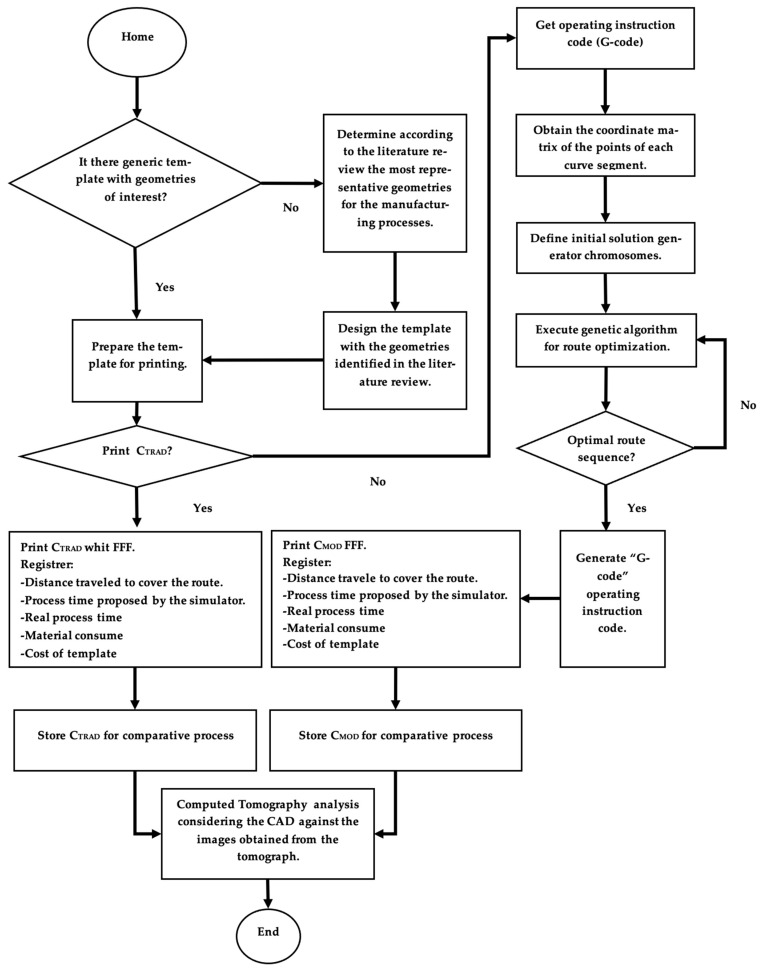
Analysis Methodology.

The present research was developed in the following stages:

1. Multi-geometric component: The template presented by Aguilar-Duque et al. [76]. The modifications consisted of the redistribution of the geometric elements and the integration of two threaded cylindrical elements.
2. Preprocessing of the multi-geometric component: The parameters described in Table 2 are used to determine the processing time required for printing the multigeometric component. The preprocessing analysis methodology is shown in Figure 1.
3. Printing of the multi-geometric component: The printing process of the control template and the template with the modified path process was carried out using an Ultimaker S5 printer. Considering the recommendations of the parameters identified in the literature review, the printing equipment was prepared according to the parameters shown in Table 3.

Polylactic acid filament (PLA) is used for the printing process. The characteristics of the material, according to the manufacturer’s datasheet, are presented in Table 4.

Before the printing process, the materials were stored in an air conditioning cabin, complying with the minimum period of 40 h, at a temperature between 23 and 25 °C until the preparation of the printing equipment.

After the printing process and to ensure that the dimensions of the components were not altered, vacuum packaging with insulation was used to prevent temperature changes and protect them from shocks. For the digitization process, the components were stored and subjected to an air-conditioning process inside a room with temperature and humidity control for a minimum period of 40 h at a temperature of 20 + 2 °C and relative humidity without condensation of 50 + 10%. The part was scanned using the ZEISS Metrotom 800 CT system for the measurement of plastic parts with high accuracy. It has a maximum spatial resolution of 3.5 μm and a Maximum Permissible Error in length measurements of (6.9 + L/100) μm and (2.9 + L/100) μm in the sphere center (L in mm).

4. Measurement of components: The components were measured using VG Studio software for porosity analyses, and Geomagic was used for dimensional analyses. Using a point cloud of the workpiece, the geometric shapes were created, and the edges of the elements were always avoided in order to avert the influence of possible imperfections. Each component was measured according to the reference coordinates. The comparative analysis considers the characteristics of interest, which are presented in Table 5.

**Figure 2:**
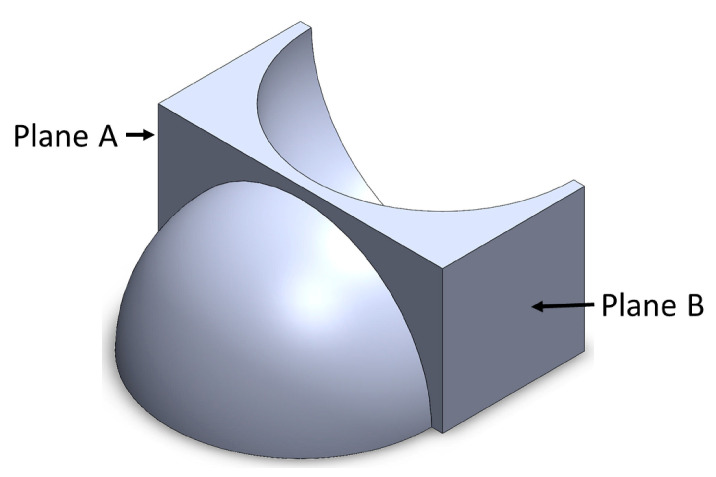
Spherical component.

The component has twenty-three geometric elements in the upper part for dimensional analyses and three geometric elements on the lateral face of the component. For each element, at least one specific attribute is described in Table 5, from which 134 attributes of interest are obtained. Figure 2 shows a component describing one attribute, which is presented in Table 6.

5. **Comparison of nominal versus actual CAD:** From the dimensions specified in the design, the error generated by the fused filament printing process is determined via tomographic analysis:
6. Comparison with the image.
7. Error calculation.
8. Classification of geometry deviations in ranges of units.
9. Classify the issue of nominal and real volumes considering the internal characteristics.

## 3. Results

### 3.1. Reduction in Manufacturing Time via GA

**Figure 3:**
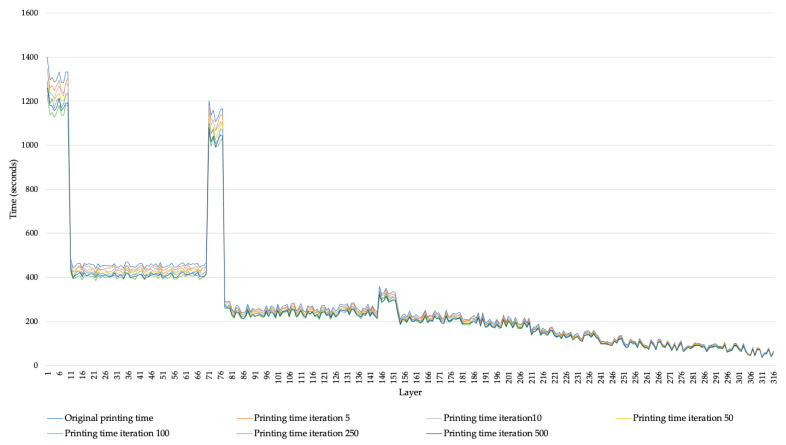
Time required per layer number.

Using the software proposed by the manufacturer, the component requires 2162 min to complete the printing process, consuming 14.34 m of material. Using the genetic algorithm to reduce the total process time required, Figure 3 shows the results of the GA by iteration, considering all the layers of the component. The selection of the layers was performed randomly.

With the modification of the tool’s path, a reduction of 11% is obtained, considering the printing time proposed by the manufacturer’s software versus the modification of the path generated by the GA. The global optimum is achieved during iteration number 94, consuming 16 min in the process of the path’s optimization and achieving a reduction in time from 2162 min to 1922 min.

### 3.2. Computed Tomography

**Figure 4:**
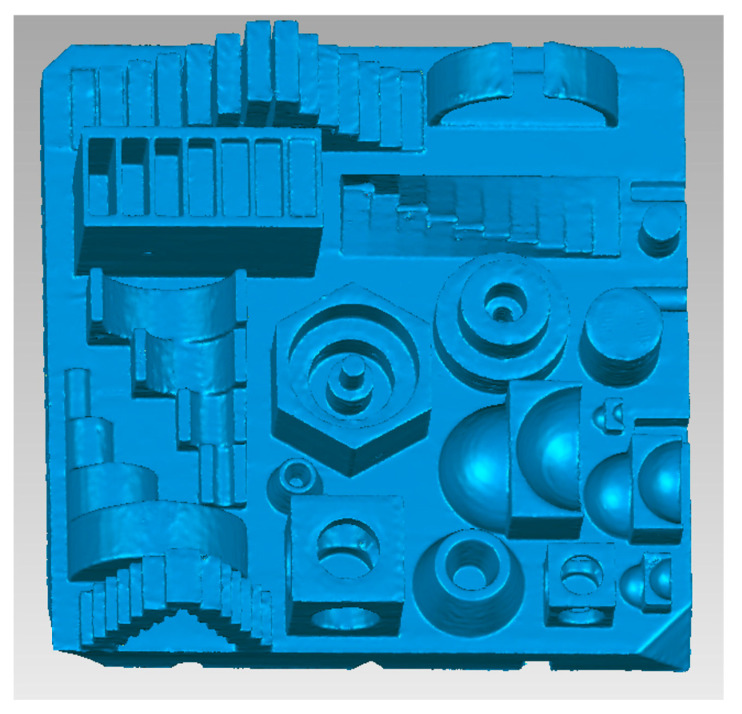
CT image of workpiece.

Figure 4 shows the 3D volume of the workpiece obtained using CT. This volume is used to perform dimensional analysis.

### 3.3. Dimensional Analysis

**Figure 5:**
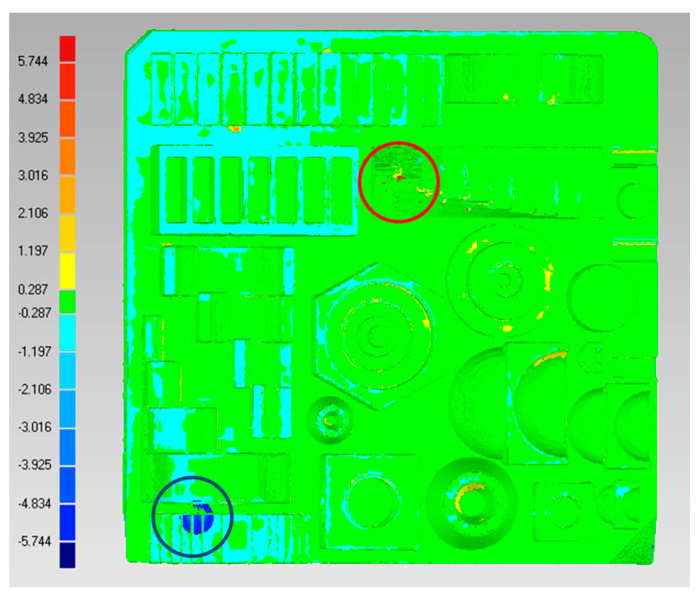
CAD comparison.

In order to obtain information to initiate a CAD comparison of the workpiece, it is possible to observe two points of maximum deviation. The points of maximum deviation are +5.57 and −5.77 mm, and they are presented in two punctual sites, as shown by the circle and red circle in Figure 5. Therefore, the average deviation of the workpiece is +0.147 and −0.12 mm.

**Figure 6:**
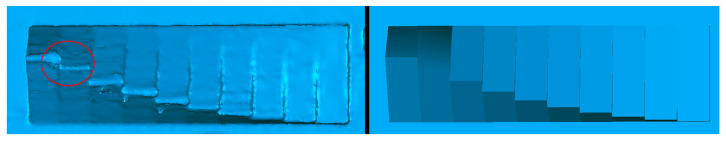
Comparison of maximum positive deviation points ((**left**) CT image, (**right**) CAD image).

Figure 6 compares the point of maximum positive deviation; it is possible to observe that the 3D printing finishes on the edges are deficient and the planes are irregular.

**Figure 7:**
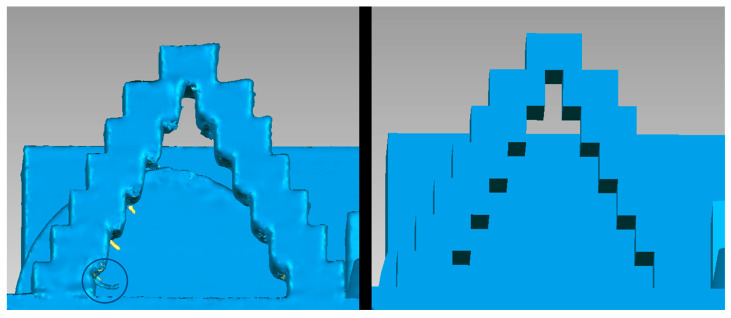
Comparison of maximum negative deviation points ((**left**) CT image, (**right**) CAD image).

Figure 7 shows the point of maximum negative deviation and its comparison against CAD. It can be seen that there are significant quality defects and extra elements compared to the original geometry, which is the main reason for the deviation against CAD.

**Figure 8:**
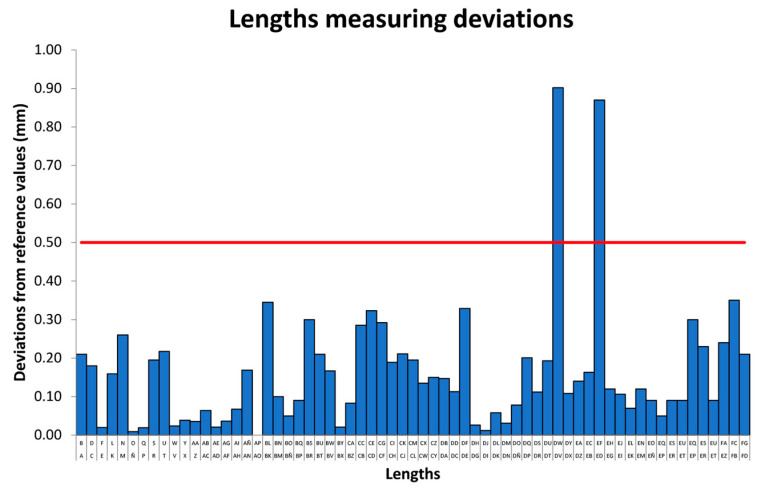
Measurement deviation (absolute values) lengths. The red line represents the fabrication tolerance.

In order to carry out a quick comparison by type of geometry, the attributes are associated with three groups: lengths, angles, and diameters. In Figure 8, it is possible to observe the absolute length deviations of the 3D volume measurement obtained with computed tomography from the original CAD design. The average deviation is 0.16 mm, the maximum is 0.90 mm, and the minimum is 0.01 mm. In this particular case, only two attributes are out of tolerance.

**Figure 9:**
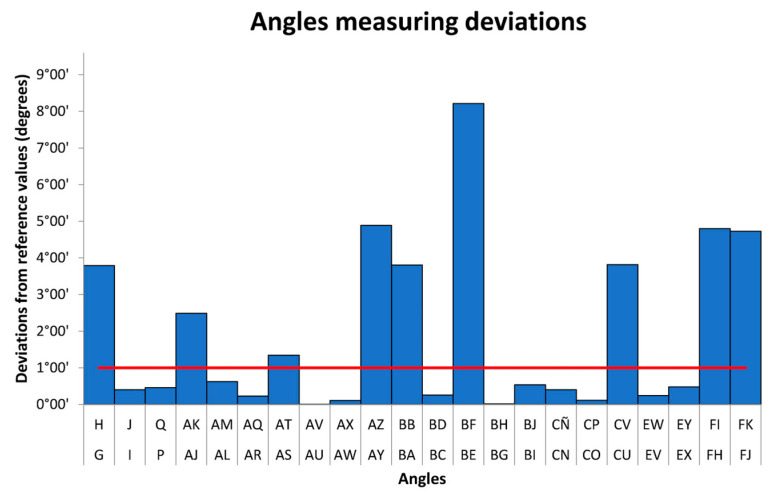
Measurement deviation (absolute values) angles. The red line represents the fabrication tolerance. The red line represents the fabrication tolerance.

In Figure 9, it is possible to observe the absolute angle’s measurement deviation. In this case, we can observe that 9 out of 22 evaluated attributes are out of tolerance, the average deviation is 1°53′, and the maximum value is 8°12′.

**Figure 10:**
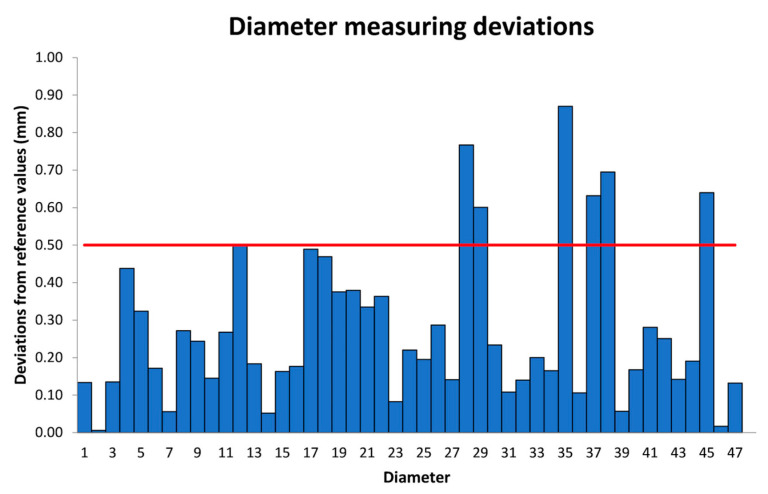
Measurement deviation (absolute values) diameters. The red line represents the fabrication tolerance.

Figure 10 shows the absolute deviation of the measurement of the group of diameters. The average deviation is 0.28 mm, the maximum value is 0.87 mm, and the minimum is 0.01 mm. In this case, 6 out of 47 attributes are out of tolerance, 5 are spherical geometries, and 1 is a half cylinder.

In summary, 17 of the 127 evaluated dimensions (Lengths, Angles, and Diameters) are out of tolerance, meaning only 13% of them do not fulfill the specification. The maximum global deviation of Lengths and Diameters is very similar, being 0.90 mm and 0.87 mm, respectively, while the average deviation of the diameters is greater than the Length by 0.12 mm. The average deviation in Angles is 1°53′.

### 3.4. Structural Analysis

**Figure 11:**
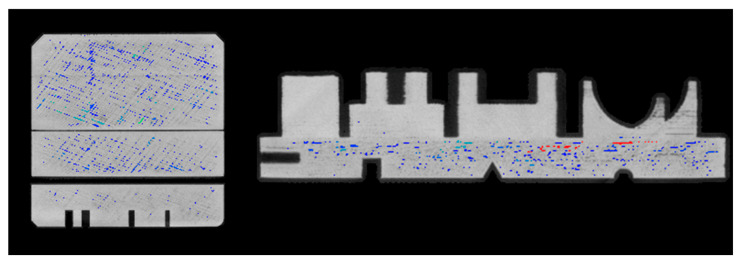
Bottom view and side view of the workpiece.

A critical parameter in additive manufacturing processes is the filling parameter, which is established at the time of printing; thus, carrying out porosity analysis is essential in order to understand the capacity of the manufacturing system. Using the software VG Studio Max from Volume Graphics, a global porosity analysis was performed. The parameter for the infill in the printed piece was 100%. In this case, the total volume of the part is 194,717.30 mm^3^, and the percentage of porosity is 3921.25 mm^3^, or 2% of the total volume; most of the porosity is at the base of the template (Figure 11).

**Figure 12:**
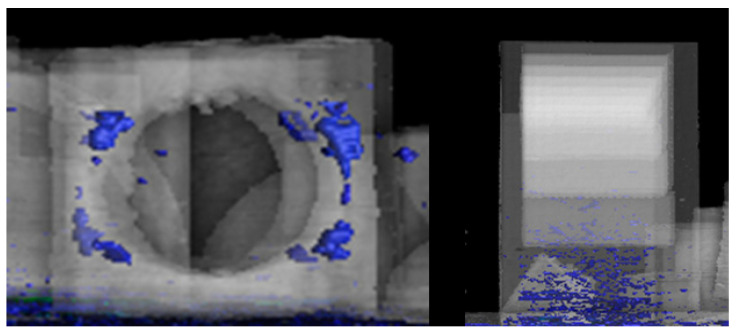
Porosity in perforated cubes and quadrangular bases.

Although the porosity of the geometries is lower than that of the base, two elements have more porosity defects in the interior than others: the perforated cube and the quadrangular base (Figure 12).

**Figure 13:**
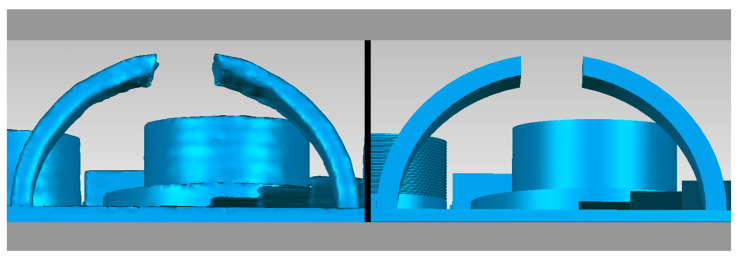
Comparison of defects in the cantilevered arc attribute ((**left**) CT image and (**right**) CAD image.

Another vital aspect of 3D printing is the surface finish and print resolution. We can appreciate some findings in the workpiece in the following figures. Figure 13 shows a quality problem in the planes of the cantilevered arch.

**Figure 14:**
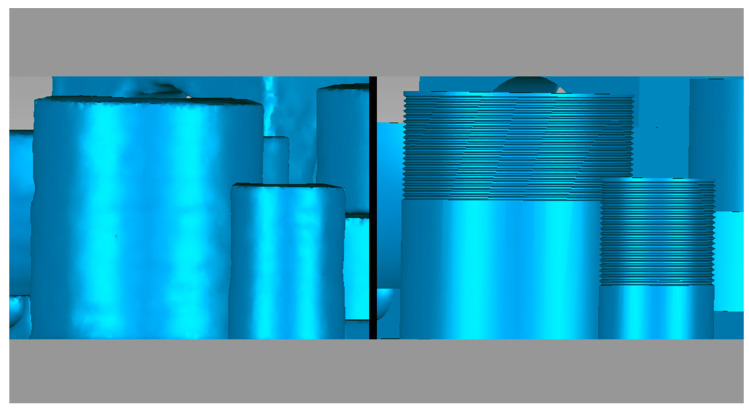
Comparison of threaded element defects ((**left**) CT image and (**right**) CAD image).

Figure 14 shows that the CT image on the left does not have the threaded element, e.g., the printer cannot print this type of geometry.

**Figure 15:**
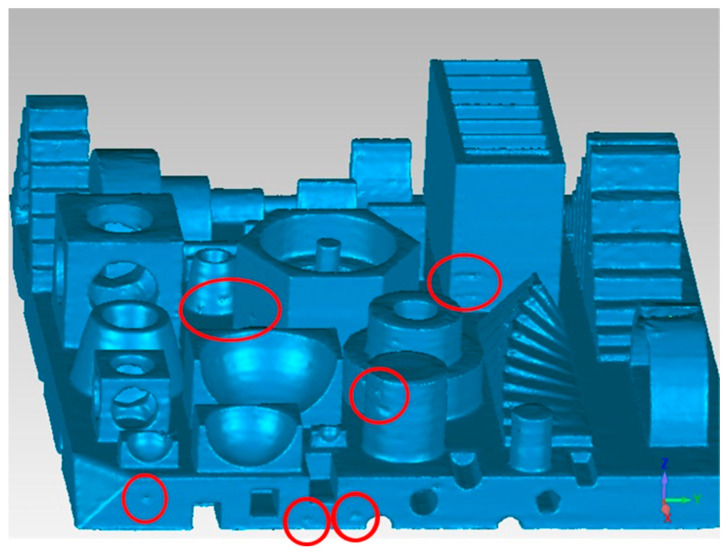
Elements with quality defects.

Figure 15 shows an isometric view of the workpiece. Some printing defects on the external faces of the geometries are shown in red circles.

## 4. Discussion

As mentioned, additive manufacturing has positioned itself in the market via personalized products. In this sense, it is important to highlight the relevance of this publication in contrast to other publications and research focused on the dimensional finish and precision that are highly linked with the efficiency of FFF in terms of printing times.

In the first instance, the procedure for modifying the printing tool path occurs, which has been shown to adapt the information used in CNC equipment via the modification of operation codes and the implementation of GA, which focuses on discriminating paths that do not modify the component and that are also not required by the deposition process.

Furthermore, in the analysis of the images and follow-up of the printing process, it is possible to define the phenomena of bulging and the seams caused by the closing of the geometry and the completion of the extrusion process. The abrupt change in the direction of the extruder generates these phenomena. It is also important to emphasize that these phenomena, which are associated with a change in direction, present the same behavior relative to various materials, in which the combination of printing temperature and deviations in the filament gauge influence the incidence of quality defects.

Referring to the step pyramid in Figure 7, as an element for the impression of dental orthoses, it is concluded that this type of geometry is not helpful for the development of implant bodies or for implant abutment, which are elements of the fastening and fixation of the dental crown. Despite being a fast and economical manufacturing process, the finish identified in the interior angles does not guarantee the fixation of the orthosis (dental crown). However, in combination with postprocessing activities, the angular components could be improved, reducing the degree of quality defects.

The uses of this technology in the automotive industry favor aesthetic elements that require specialized molds and that will not be exposed to mechanical stresses. In the case of inclined planes, it is possible to identify that this technology is not useful for the determination of gears that require precision relative to circular pitch, tooth thickness, ridge, face, shoulder, and the valley of the toothed element. However, structurally, the printing process of angled elements is acceptable due to its low porosity; thus, the technology is recommended under the restriction of the magnitude of the angle.

## 5. Conclusions

GA in reducing tool path time is one of many strategies employed by technologies based on the principle of computer numerical control (CNC), such as fused filament manufacturing. By modifying the path, it is possible to reduce the time required for manufacturing by 11%, from 2162 min to 1922 min.

Concerning the dimensional finish, it is observed that 3 out of the 23 components considered in the template suffered effects on the dimensional finish, which are associated with phenomena related to the modification of the extruder’s path and deviations with respect to the diameter of the material. The affected components are the inclined planes, the cantilevered arch, and the stepped pyramid, which, due to their characteristics, presented variations in the vertices generated by the planes.

Finally, it should be noted that the characteristics identified with CT rendered the analysis of cylindrical elements, inclined planes for angle measurements, and the cantilevered arch in the dimensional finishing and shape definition possible. In them, maximum deviations were identified in the CAD comparison as +5.57 and −5.77 mm caused by foreign bodies, which are outliers caused by defects in the fabrication process.

## Figures and Tables

**Table 1:** Production sectors with the greatest development within the manufacturing industry [10].

Industry	Developed
Automotive	644
Chemicals	520
Building materials	180
Textiles and Clothing	112
Pharmaceuticals	317
Precision Instruments	185
Machine Tools	175
Aerospace	50

**Table 2:** Operating parameters of the FFF printing equipment.

Parameter	Magnitude	Reference
Nozzle diameter	0.3 mm	[77]
Quality	Magnitude	[78,79]
Layer height	0.15 mm	
Perimeter	Magnitude	[79,80]
Wall thickness	0.8 mm	
Upper/lower thickness	0.8 mm	
Filling	Magnitude	[15,81]
Filling density	100%	
Filling pattern	Lines	
Material	Magnitude	[82,83]
Printing temperature	215 °C	
Printing bed temperature	60 °C	
Diameter	1.75 mm	
Flow	100%	
Retraction	Enabled	
Speed	Magnitude	[15,79]
Printing speed	60 mm/s	
Travel speed	120 mm/s	
Cooling	Magnitude	[84]
Print cooling	Activate	
Support	Magnitude	[85,86,87,88]
Generate support	Active for -Z	
Support placement	Everywhere	
Adhesion of the printing plate		[77,85,86,89]
Type of adhesion	Border	
Edge width	8.00 mm	

**Table 3:** Printing equipment characteristics.

Feature	Technical Data
Print size	330 × 240 × 300 mm
Feeding	100–240 VAC, 50–60 Hz
Software	Ultimaker Cura
XYZ Resolution	6.9, 6.9, 2.5 micras
Nozzle diameter	0.25
Nozzle temperature	180–260 °C
Bed temperature	20–110 °C
Printing speed	<24 mm^3^/s

**Table 4:** PLA characteristics.

Characteristics	Technical Data
Appearance	Filament
Color	White
Ignition temperature	388 °C
Thermal decomposition	250 °C
Melting point/melting range	145–160 °C
Density	1.24 g/cm^3^

**Table 5:** Attributes for comparison between components.

Feature	Geometry
Flat base	Quadrangular base, set of blocks, pyramid overhang.
Prisms	Base quadrangular groove, base quadrangular groove, perforated cube, ladder, grooves.
Cylindrical drilling	Coaxial cylinders, base pass-through drilling, base drilling, and cube-drills.
Sphere	Set of spheres.
Solid cylinder	Concave semi-cylinders.
Hollow cylinder	Convex semi-cylinders, cantilevered arch.
Cone	Truncated cones.
Angled surfaces	Truncated cones, triangular perforations, staircases, inclined planes, and pyramids of blocks.

**Table 6:** Specifications for attribute measurement.

Magnitude	Feature	Description	Reference Value
Long	Plane A to Plane B	Distance from Plane A to Plane B (Plane A is parallel and opposite to Plane B)	13 + 0.5 mm

## Data Availability

The data presented in this study are available on request from the corresponding author.
